# Shock Wave Therapy Enhances Mitochondrial Delivery into Target Cells and Protects against Acute Respiratory Distress Syndrome

**DOI:** 10.1155/2018/5425346

**Published:** 2018-10-21

**Authors:** Kun-Chen Lin, Christopher Glenn Wallace, Tsung-Cheng Yin, Pei-Hsun Sung, Kuan-Hung Chen, Hung-I Lu, Han-Tan Chai, Chih-Hung Chen, Yi-Ling Chen, Yi-Chen Li, Pei-Lin Shao, Mel S. Lee, Jiunn-Jye Sheu, Hon-Kan Yip

**Affiliations:** ^1^Department of Anesthesiology, Kaohsiung Chang Gung Memorial Hospital and Chang Gung University College of Medicine, Kaohsiung 83301, Taiwan; ^2^Department of Plastic Surgery, University Hospital of South Manchester, Manchester, UK; ^3^Department of Orthopaedic Surgery, Kaohsiung Chang Gung Memorial Hospital and Chang Gung University College of Medicine, Kaohsiung 83301, Taiwan; ^4^Center for Shockwave Medicine and Tissue Engineering, Kaohsiung Chang Gung Memorial Hospital, Kaohsiung 83301, Taiwan; ^5^Division of Cardiology, Department of Internal Medicine, Kaohsiung Chang Gung Memorial Hospital and Chang Gung University College of Medicine, Kaohsiung 83301, Taiwan; ^6^Division of Thoracic and Cardiovascular Surgery, Department of Surgery, Kaohsiung Chang Gung Memorial Hospital and Chang Gung University College of Medicine, Kaohsiung 83301, Taiwan; ^7^Divisions of General Medicine, Department of Internal Medicine, Kaohsiung Chang Gung Memorial Hospital and Chang Gung University College of Medicine, Kaohsiung 83301, Taiwan; ^8^Institute for Translational Research in Biomedicine, Kaohsiung Chang Gung Memorial Hospital, Kaohsiung 83301, Taiwan; ^9^Department of Nursing, Asia University, 500 Lioufeng Rd., Wufeng, Taichung 41354, Taiwan; ^10^Department of Medical Research, China Medical University Hospital, China Medical University, Taichung 40402, Taiwan

## Abstract

This study tested the hypothesis that shock wave therapy (SW) enhances mitochondrial uptake into the lung epithelial and parenchymal cells to attenuate lung injury from acute respiratory distress syndrome (ARDS). ARDS was induced in rats through continuous inhalation of 100% oxygen for 48 h, while SW entailed application 0.15 mJ/mm^2^ for 200 impulses at 6 Hz per left/right lung field. In vitro and ex vivo studies showed that SW enhances mitochondrial uptake into lung epithelial and parenchyma cells (all *p* < 0.001). Flow cytometry demonstrated that albumin levels and numbers of inflammatory cells (Ly6G+/CD14+/CD68+/CD11^b/c^+) in bronchoalveolar lavage fluid were the highest in untreated ARDS, were progressively reduced across SW, Mito, and SW + Mito (all *p* < 0.0001), and were the lowest in sham controls. The same profile was also seen for fibrosis/collagen deposition, levels of biomarkers of oxidative stress (NOX-1/NOX-2/oxidized protein), inflammation (MMP-9/TNF-*α*/NF-*κ*B/IL-1*β*/ICAM-1), apoptosis (cleaved caspase 3/PARP), fibrosis (Smad3/TGF-*β*), mitochondrial damage (cytosolic cytochrome c) (all *p* < 0.0001), and DNA damage (*γ*-H2AX+), and numbers of parenchymal inflammatory cells (CD11+/CD14+/CD40L+/F4/80+) (*p* < 0.0001). These results suggest that SW-assisted Mito therapy effectively protects the lung parenchyma from ARDS-induced injury.

## 1. Introduction

The respiratory system (i.e., oral-nasal-laryngeal tract, bronchus, trachea, and alveolar sacs in the lungs) is critical for maintenance of an adequate oxygen supply and excretion of carbon dioxide (CO_2_). For this purpose, the lung parenchyma has a dense capillary network mediating gaseous exchange. The lung is vulnerable to damage from a variety of causes, including viruses, bacteria, toxic chemicals/smoke, food aspiration, septic or cardiogenic shock, resuscitation after circulatory arrest, alveolar-type pulmonary edema, massive blood transfusion, and ischemia-reperfusion injury after bypass surgery or organ transplantation [1–9]. Moreover, sustained insults may ultimately develop into clinical acute respiratory distress syndrome (ARDS) [9–11]. Despite pharmacologic advances and continuous renewal of management strategies [12–16], in-hospital mortality from ARDS remains unacceptably high [9, 10, 17–19]. Consequently, there is an urgent need for a safe and efficacious alternative treatment for this high-risk group of patients.

The mechanisms underlying acute lung injury/ARDS are multifactorial and include inflammation, alveolar leukocytosis, protein leakage, mitochondrial-free radical production, mitochondrial damage/loss, generation of reactive oxygen species (ROS) and resultant lung oxidant stress, and apoptosis [4, 9, 20–24]. Within this context, we hypothesize that a treatment that could reverse mitochondrial loss may, in turn, inhibit ROS-free radical generation and improve ARDS parameters. Previous studies by others [25–27] and ourselves [28–30] have shown that mitochondrial transfusion effectively protects against acute organ damage, including sepsis-induced acute lung injury [13, 25], acute ischemia-reperfusion injury of the heart [27], liver [26, 28], and lung [30], and monocrotaline-induced pulmonary arterial hypertension [29]. Additionally, shock wave (SW) therapy entailing delivery of a series of transient pressure waves characterized by a high peak pressure (100 MPa), fast pressure rise (<10 ns), rapid propagation, and short life cycle (10 *μ*s) produced by an appropriate generator reportedly reverses ischemia-related organ dysfunction, mainly by enhancing angiogenesis, recruiting endothelial progenitor cells, and suppressing inflammation and oxidative stress [31–33]. In the present study, therefore, we used a rat model to test the hypothesis that SW-assisted mitochondrial therapy would be superior to either therapy alone for protection of the lung against ARDS injury.

## 2. Materials and Methods

### 2.1. Ethics

All animal experimental procedures were approved by the Institute of Animal Care and Use Committee at Kaohsiung Chang Gung Memorial Hospital (Affidavit of Approval of Animal Use Protocol number 2016032205) and performed in accordance with the Guide for the Care and Use of Laboratory Animals (The Eighth Edition of the Guide for the Care and Use of Laboratory Animals (NRC 2011)).

Animals were housed in an Association for Assessment and Accreditation of Laboratory Animal Care International- (AAALAC-) approved animal facility in our hospital with controlled temperature and light cycle (24°C and 12/12 light cycle).

### 2.2. Inducing ARDS in SD Rats

The ARDS model used in this study has been described in our recent studies [30, 34], wherein pure oxygen (i.e., 100% O_2_) was continuously administered to the rat for 48 h. In detail, a close system of glass (i.e., for monitoring the safety of each animal) square box was created. Inside the close system, the adequate food and water were provided for the animals. At least five animals were accommodated for each time in the glass box. Oxygen cannulation was firmly connected to the glass box with an oxygen meter to monitor the oxygen gas supply in the box to achieve each animal exposure to 100% oxygen for 48 hours.

### 2.3. Animal Grouping and Application of SW

Pathogen-free, adult male Sprague-Dawley (SD) rats (*n* = 30) weighing 325–350 g (Charles River Technology, BioLASCO, Taiwan Co. Ltd., Taiwan) were randomized into five groups (*n* = 6 in each group): group 1, sham controls (intravenous injection of 0.5 ml of normal saline); group 2, ARDS; group 3, ARDS + SW (0.15 mJ/mm^2^ for 200 impulses at 6 Hz per left/right lung field applied once 3 h after completing 48 h of oxygen inhalation); group 4, ARDS + mitochondria (Mito) (2000 *μ*g/rat administered intravenously with a time interval identical to SW therapy); and group 5, ARDS + SW + Mito. Animals were sacrificed on day 5 after ARDS induction.

All animals were anesthetized (inhaled 2.0% isoflurane) in a supine position on a warming pad at 37°C during the application of SW to the left and then right lungs. To avoid affecting the heart during SW, the focus type of SW (Storz Duolith SD1, STORZ MEDICAL AG, Switzerland) was utilized. In addition, mitochondrial oxygen consumption rates were determined using a Mito stress test kit (i.e., Seahorse Bioscience, Billerica, MA) and the XF24 Analyzer.

### 2.4. Mitochondrial Isolation from Donors and MitoTracker Staining for Mitochondria

Liver mitochondria were isolated from donor SD rats as previously described [35]. The rats were fasted overnight prior to the mitochondrial isolation procedure, then sacrificed, and their gallbladders and livers were carefully removed. Immediately, the liver (3 g) was immersed in 50 ml of ice-cold IBc (10 mM Tris-MOPS, 5 mM EGTA/Tris, 200 mM sucrose, and pH 7.4.) in a beaker, followed by rinsing the liver free of blood with ice-cold IBc. The liver was then minced with scissors in a beaker surrounded by ice. IBc was discarded during mincing and replaced with 18 ml of ice-cold fresh IBc. The liver was then homogenized with a Teflon pestle. The homogenates were transferred to a 50 ml polypropylene Falcon tube and centrifuged at 600*g* for 10 minutes at 4°C. The supernatants were transferred to fresh tubes for centrifugation at 7000*g* for 10 minutes at 4°C. The supernatants were discarded, and the pellets were washed with 5 ml ice-cold IBc. Again, the supernatants from the pellets were centrifuged at 7000*g* for 10 minutes at 4°C. The supernatants were discarded, and the pellets containing the mitochondria were resuspended. The concentration of the mitochondrial suspensions was measured using the Biuret method. Each 10 mg of isolated mitochondria was labeled with 1 M of MitoTracker Red CMXRos (Invitrogen, Carlsbad, CA) through incubation at 37°C for 30 minutes. Mitochondrial transfusion was performed for the study animals immediately after labeling (i.e., <3 hrs after the isolation procedure).

### 2.5. Procedure and Protocol for Quantification of Oxygen Consumption Rate (OCR) of Isolated Mitochondria (Seahorse Method) (Figure 1)

The procedure and protocol have also been described in our recent report [30]. In detail, functional activity of isolated mitochondria from rat liver was determined with an Extracellular Flux Analyzer (XF^e^24, Seahorse Bioscience, MA, USA) by assessing the degree of coupling between the electron transport chain (ETC) and the oxidative phosphorylation machinery (OXPHOS). Bioenergetics of mitochondria as reflected in the integrity of electron transport chain and capacity of oxidative phosphorylation were evaluated by measuring the mitochondrial oxygen consumption rate (OCR). In this study, isolated mitochondria (10 *μ*g/well) from rat liver were diluted in cold 1X mitochondrial assay solution (MAS) (70 mM sucrose, 220 mM mannitol, 10 mM KH_2_PO_4_, 5 mM MgCl_2_, 2 mM HEPES, 1.0 mM EGTA, and pH 7.2), followed by spinning down at 3000*g* for 30 minutes. After attachment of mitochondria to XF24 plate, coupling reaction was initiated with the administration of substrate (10.0 mM succinate). State 3 was initiated with ADP (0.5 mM), while state 4 was induced with the addition of oligomycin (2 *μ*M). Maximal uncoupler-stimulated respiration was elicited with FCCP (4 *μ*M), whereas complex III repression was induced by antimycin A (4 *μ*M). OCR of mitochondria in reactions mentioned above was sequentially measured.

### 2.6. In Vitro Study for Determining the Impact of SW on Enhancing Mitochondrial Transfusion in the Rat Lung Epithelial Cells (LEC)

To elucidate whether SW therapy could enhance mitochondrial transfusion into the rat lung epithelial cells (LEC), the LECs were cultured in F-12K medium (i.e., 10% FBS + 1% Penicillin-Streptomycin (Gibco) in 1 × 10^6^ cells) in T25 flask for 24 h, followed by with and without SW treatment (0.2 mJ/mm^2^ for 100 shots). The cells (3 × 10^4^) were then cultured in EZ slide. 24 h after the cell culturing, 50 *μ*g of exogenous mitochondria was transfused into the cultured cells (3 × 10^4^). 90 minutes later after transfusion, the endogenous mitochondria in these cells were stained by MitoTracker Green (Invitrogen M7514, 1 : 100 nM). Additionally, these cells were also stained by MitoTracker Orange (Invitrogen M7510, 1 : 500 nM).

### 2.7. Pathological Assessment of Lung Injury

The procedure and protocol have been described in our previous reports [23, 24, 30, 35]. In detail, lung specimens were sectioned at 5 *μ*m for light microscopy and H&E staining was performed to investigate the number of alveolar sacs in a blinded fashion [23, 24, 30, 35]. Three lung sections from each rat were analyzed, and three randomly selected high-power fields (HPFs; 200x) were examined in each section. The mean number per HPF for each animal was then determined by a summation of all numbers divided by 9. The extent of crowded area, which was defined as the region of thickened septa in lung parenchyma associated with partial or complete collapse of alveoli on H&E-stained sections, was also performed in a blinded fashion. The following scoring system [23, 24] was adopted: 0 = no detectable crowded area; 1 = <15% of crowded area; 2 = 15–25% of crowded area; 3 = 25–50% of crowded area; 4 = 50–75% of crowded area; 5 = >75%–100% of crowded area/HPF.

### 2.8. Bronchoalveolar Lavage and Lung Specimen Preparation

To elucidate the impact of SW mitochondrial treatment on protecting the lung from ARDS injury, bronchoalveolar lavage (BAL) was performed and the BAL fluid was collected for study in six additional rats from each group.

### 2.9. Immunofluorescent (IF) Studies

The procedures and protocols for IF examination were also based on our recent study [23, 24, 30, 35]. Briefly, for IF staining, rehydrated paraffin sections were first treated with 3% H_2_O_2_ for 30 minutes and incubated with Immuno-Block reagent (BioSB, Santa Barbara, CA, USA) for 30 minutes at room temperature. Sections were then incubated with primary antibodies specifically against CD14 (1 : 50, Santa Cruz), CD11 (1 : 500, Abcam), F4/80 (1 : 100, Santa Cruz), CD40L (1 : 100, Abcam), and *γ*-H2AX (1 : 1000, Abcam) while sections incubated with irrelevant antibodies served as controls. Three kidney sections from each rat were analyzed. For quantification, three randomly selected HPFs (400x for IF) were analyzed in each section. The mean number of positively stained cells per HPF for each animal was then determined by the summation of all numbers divided by 9.

### 2.10. Western Blot Analysis

The procedure and protocol for Western blot analysis have been described in our previous reports [23, 24, 30, 35]. Briefly, equal amounts (50 *μ*g) of protein extract were loaded and separated by SDS-PAGE using acrylamide gradients. After electrophoresis, the separated proteins were transferred electrophoretically to a polyvinylidene difluoride (PVDF) membrane (Amersham Biosciences, Amersham, UK). Nonspecific sites were blocked by incubation of the membrane in blocking buffer (5% nonfat dry milk in T-TBS (TBS containing 0.05% Tween 20)) overnight. The membranes were incubated with the indicated primary antibodies (matrix metalloproteinase- (MMP-) 9 (1 : 3000, Abcam, ab76003, Cambridge, MA, USA), tumor necrosis factor- (TNF-) *α* (1 : 1000, Cell Signaling, number 3707, Danvers, MA, USA), nuclear factor- (NF-) *κ*B p65 (1 : 600, Abcam, ab16502, Cambridge, MA, USA), NADPH oxidase- (NOX-) 1 (1 : 1500, Sigma, SAB4200097, St. Louis, Mo, USA), NOX-2 (1 : 750, Sigma, SAB4200118, St. Louis, Mo, USA), interleukin- (IL-) 1*β* (1 : 1000, Cell Signaling, number 12426, Danvers, MA, USA), intercellular adhesion molecule- (ICAM-) 1 (1 : 1000, Abcam, ab2213, Cambridge, MA, USA), caspase 3 (1 : 1000, Cell Signaling, number 9665, Danvers, MA, USA), cleaved poly (ADP-ribose) polymerase (c-PARP) (1 : 1000, Cell Signaling, number 9542), transforming growth factor- (TGF-) *β* (1 : 500, Abcam, ab64715), phosphorylated- (p-) Smad3 (1 : 1000, Cell Signaling, number 9520), cytosolic cytochrome c (1 : 1000, BD, 556433, Franklin, NJ, USA), mitochondrial cytochrome c (1 : 1000, BD, 556433, Franklin, NJ, USA), and actin (1 : 10000, Millipore, number MAB1501, Billerica, MA, USA)) for 1 hour at room temperature. Horseradish peroxidase-conjugated anti-rabbit immunoglobulin IgG (1 : 2000, Cell Signaling, number 7074, Danvers, MA, USA) was used as a secondary antibody for a one-hour incubation at room temperature. The washing procedure was repeated eight times within one hour. Immunoreactive bands were visualized by enhanced chemiluminescence (ECL; Amersham Biosciences, Amersham, UK) and exposed to Biomax L film (Kodak, Rochester, NY, USA). For quantification, ECL signals were digitized using Labwork software (UVP, Waltham, MA, USA).

### 2.11. Assessment of Oxidative Stress

The procedure and protocol for assessing the protein expression of oxidative stress have been detailed in our previous reports [23, 24, 30, 35]. The Oxyblot Oxidized Protein Detection Kit was purchased from Chemicon, Billerica, MA, USA (S7150). DNPH derivatization was carried out on 6 *μ*g of protein for 15 minutes according to the manufacturer's instructions. One-dimensional electrophoresis was carried out on 12% SDS-polyacrylamide gel after DNPH derivatization. Proteins were transferred to nitrocellulose membranes which were then incubated in the primary antibody solution (anti-DNP 1 : 150) for 2 hours, followed by incubation in secondary antibody solution (1 : 300) for 1 hour at room temperature. The washing procedure was repeated eight times within 40 minutes. Immunoreactive bands were visualized by enhanced chemiluminescence (ECL; Amersham Biosciences, Amersham, UK) which was then exposed to Biomax L film (Kodak, Rochester, NY, USA). For quantification, ECL signals were digitized using Labwork software (UVP, Waltham, MA, USA). For Oxyblot protein analysis, a standard control was loaded on each gel.

### 2.12. Histopathological Assessment in Lung Parenchyma

To analyze the integrity of collagen synthesis and deposition, three lung paraffin sections (4 *μ*m) were stained with Picrosirius red (1% Sirius red in saturated picric acid solution) for one hour at room temperature using standard methods. The sections were then washed twice with 0.5% acetic acid. The water was physically removed from the slides by vigorous shaking. After dehydration in 100% ethanol thrice, the sections were cleaned with xylene and mounted in a resinous medium. Ten low-power fields (10x) of each section were used to identify Sirius red-positive area on each section. The integrated area (*μ*m^2^) of condensed collagen deposition in each section was calculated using Image Tool 3 (IT3) image analysis software (University of Texas, Health Science Center, San Antonio (UTHSCSA); Image Tool for Windows, Version 3.0, USA). Three selected sections were quantified for each animal. Three randomly selected HPFs (100x) were analyzed in each section. After determining the number of pixels in each collagen deposition area per HPF, the numbers of pixels obtained from the three HPFs were summed. The procedure was repeated in two other sections for each animal. The mean pixel number per HPF for each animal was then determined by summing all pixel numbers and divided by 9. The mean integrated area (*μ*m^2^) of collagen deposition area in lung parenchyma per HPF was obtained using a conversion factor of 19.24 (1 *μ*m^2^ corresponded to 19.24 pixels). To elucidate the fibrosis of lung parenchyma, Masson's trichrome stain was performed according to the manufacturer's instruction. The analytical method for identification of fibrotic area was identical to the method for analysis of condensed collagen deposition area.

### 2.13. Statistical Analysis

Quantitative data are expressed as means ± SD. Statistical analysis was adequately performed by ANOVA followed by Bonferroni multiple comparison post hoc test. Statistical analysis was performed using SPSS statistical software for Windows version 22 (SPSS for Windows, version 22; SPSS, IL, USA). A value of *p* < 0.05 was considered as statistically significant.

## 3. Results

### 3.1. SW Therapy Enhances Exogenous Mitochondrial Transfusion into Lung Epithelial Cells

**Figure 1:**
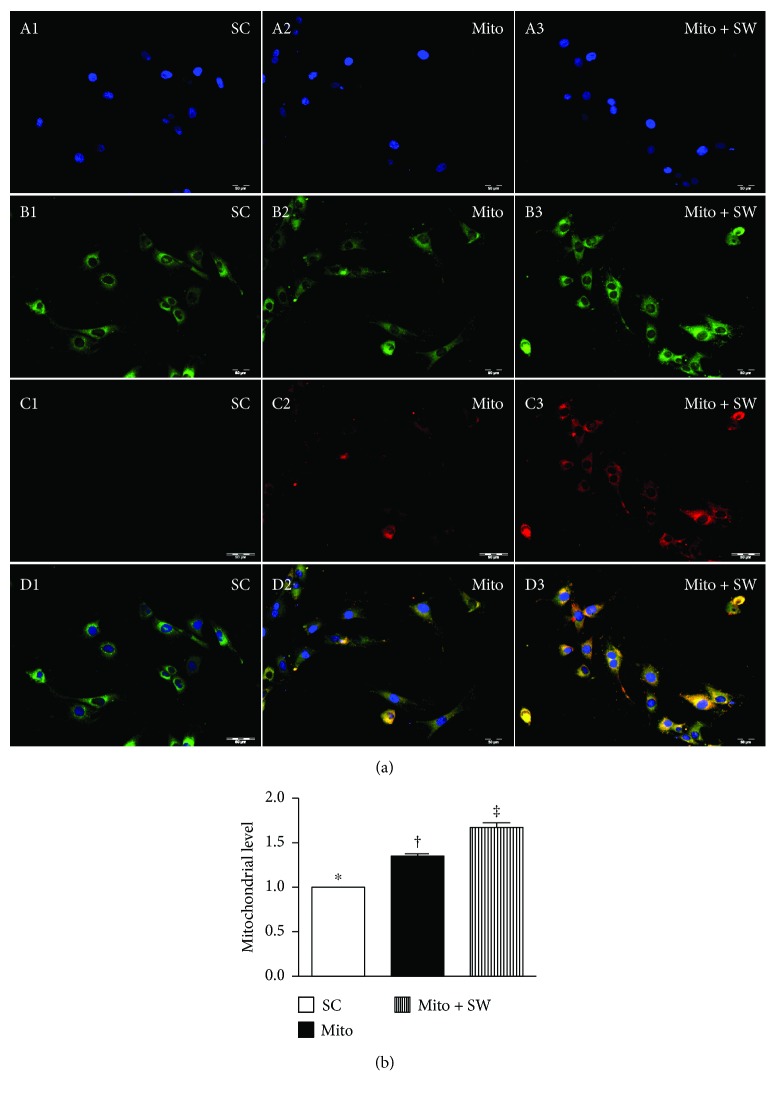
In vitro study showed SW therapy enhanced mitochondrial transfusion into the rat lung epithelial cells. (A1 to A3) Illustrating the DAPI stain (400x) for identification of rat lung epithelial cells in three groups (i.e., SC, Mito, and Mito + SW). (B1 to B3) Showing MitoTracker stain (400x) for identification of endogenous mitochondria (green color) among three groups. (C1 to C3) Indicating the MitoTracker stain (400x) for identification of exogenous mitochondria to be transfused into the epithelial cells (red color). (D1 to D3) Indicating the merged pictures of B1–B3 and C1–C3. Pink-yellow color indicated that the endogenous and exogenous mitochondria colocalized together. An abundant number of mitochondria were found in the Mito + SW group. (b) Analytic result of mitochondrial level in the cells, ^∗^ versus other groups with different symbols (†, ‡), *p* < 0.001. All statistical analyses were performed by one-way ANOVA, followed by Bonferroni multiple comparison post hoc test (*n* = 6 for each group). Symbols (^∗^, †, ‡) indicate significance (at 0.05 level). SC = sham control; Mito = mitochondria; SW = shock wave.

**Figure 2:**
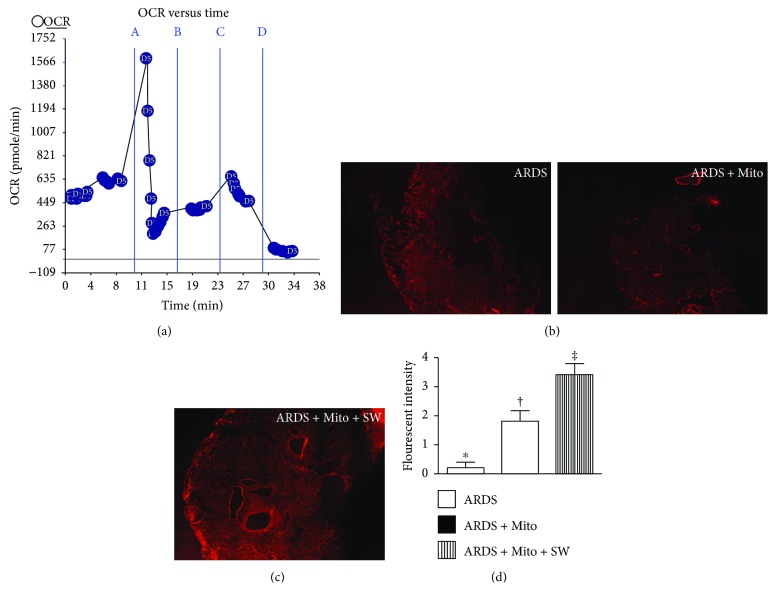
Mitochondrial functional assay and in vivo showed SW therapy enhanced mitochondrial transfusion into the cells. (a) Mitochondrial functional assay showed a satisfactory activity (i.e., high oxygen consumption rate) of isolated mitochondria (determined by the Mito stress test kit and the XF^e^24 Analyzer) (*n* = 4). (b, c) Illustrating the confocal findings (400x) of lung specimen at a time interval of 24 h after intravenously mitochondrial transfusion. (d) Analytical results of fluorescent intensity, ^∗^ versus other groups with different symbols (†, ‡), *p* < 0.001. All statistical analyses were performed by one-way ANOVA, followed by Bonferroni multiple comparison post hoc test (*n* = 6 for each group). Symbols (^∗^, †, ‡) indicate significance (at 0.05 level). OCR = oxygen consumption rate. ARDS = acute respiratory distress syndrome; Mito = mitochondria; SW = shock wave.

In vitro observations revealed that SW therapy significantly enhanced transfusion of exogenous mitochondria into rat lung epithelial cells as compared to transfusion alone (Figure 1). In vivo, numbers of mitochondria in the lung parenchyma of ARDS rats treated with SW plus mitochondrial transfusion (SW + Mito) were significantly higher than those in the lungs of untreated ARDS animals or ARDS animals treated with Mito alone (Figure 2). Measurement of the mitochondrial oxygen consumption rate showed that mitochondrial activity was satisfactory for purposes of the present study (Figure 2).

### 3.2. Flow Cytometric Analysis of Inflammatory Mediators and Albumin Leakage in Bronchoalveolar Lavage (BAL) Fluid

**Figure 3:**
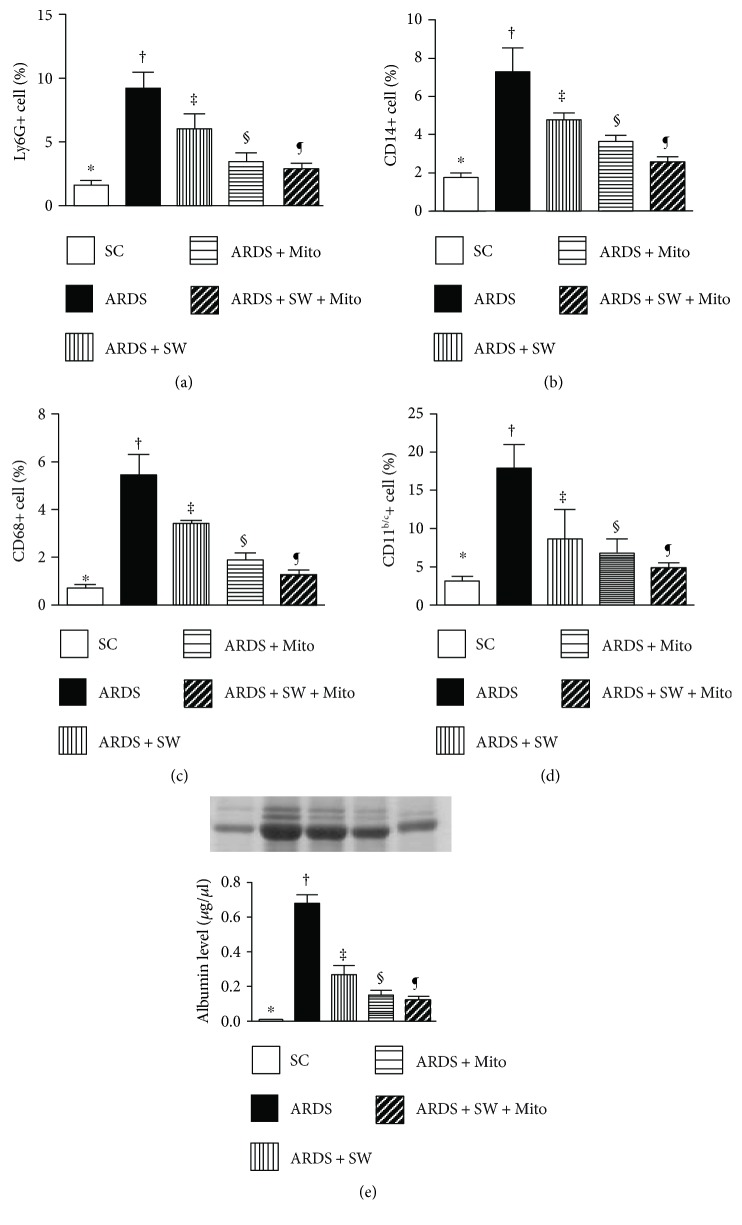
Inflammatory mediators and leakage of albumin in bronchoalveolar lavage (BAL) fluid by day 5 after ARDS induction. (a) Flow cytometric analysis of numbers of Ly6G+ cells in BAL, ^∗^versus other groups with different symbols (†, ‡, §, ¶), *p* < 0.0001. (b) Flow cytometric analysis of numbers of CD14+ cells in BAL, ^∗^versus other groups with different symbols (†, ‡, §, ¶), *p* < 0.0001. (c) Flow cytometric analysis of numbers of CD68+ cells in BAL, ^∗^versus other groups with different symbols (†, ‡, §, ¶), *p* < 0.0001. (d) Flow cytometric analysis of numbers of CD11^b/c^+ cells in BAL, ^∗^versus other groups with different symbols (†, ‡, §, ¶), *p* < 0.0001. (e) Protein expression of albumin level in BAL, ^∗^versus other groups with different symbols (†, ‡, §, ¶), *p* < 0.0001. All statistical analyses were performed by one-way ANOVA, followed by Bonferroni multiple comparison post hoc test (*n* = 6 for each group). Symbols (^∗^, †, ‡, §, ¶) indicate significance (at 0.05 level). SC = sham control; ARDS = acute respiratory distress syndrome; Mito = mitochondria; SW = shock wave.

Flow cytometric analysis demonstrated that the numbers of Ly6G+, CD14+, CD68+, and CD11^b/c^+ inflammatory cells were highest in BAL fluid from untreated ARDS rats, were significantly and progressively lower across the ARDS + SW, ARDS + Mito, and ARDS + SW + Mito groups, and were the lowest in the sham control (SC) group (Figure 3). In addition, Western blot analysis showed that the profile of albumin levels in BAL fluid, an indicator of increased lung permeability and exudate leakage related to lung parenchymal damage in ARDS, exhibited the same pattern as the inflammatory cells among the five groups.

### 3.3. Pathological Findings after ARDS Induction

**Figure 4:**
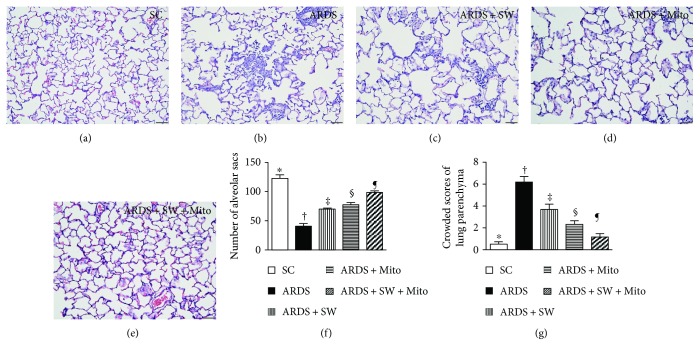
Pathological findings of lung parenchyma by day 5 after ARDS induction. (a to e) Pathological findings (i.e., H&E staining) of lung parenchyma under microscopy (200x) among the five groups. The scale bars in the right lower corner represent 50 *μ*m. (f) The number of alveolar sacs among five groups. ^∗^ versus other groups with different symbols (†, ‡, §, ¶), *p* < 0.0001. (g) Crowded scores of lung parenchyma. ^∗^ versus other groups with different symbols (†, ‡, §, ¶), *p* < 0.0001. All statistical analyses were performed by one-way ANOVA, followed by Bonferroni multiple comparison post hoc test. Symbols (^∗^, †, ‡, §, ¶) indicate significance (at 0.05 level). SC = sham control; ARDS = acute respiratory distress syndrome; Mito = mitochondria; SW = shock wave.

Hematoxylin and eosin staining revealed that 5 days after ARDS induction, numbers of alveolar sacs, an index of lung parenchyma integrity, was the lowest in untreated ARDS, increased progressively and significantly across the ARDS + SW, ARDS + Mito, and ARDS + SW + Mito groups, and was the highest in the SC group. Conversely, scoring of lung parenchymal crowding, an index of lung parenchymal damage, showed the opposite pattern in the five groups (Figure 4).

### 3.4. Inflammatory Cell Infiltration of Lung Parenchyma and DNA Damage after ARDS Induction

**Figure 5:**
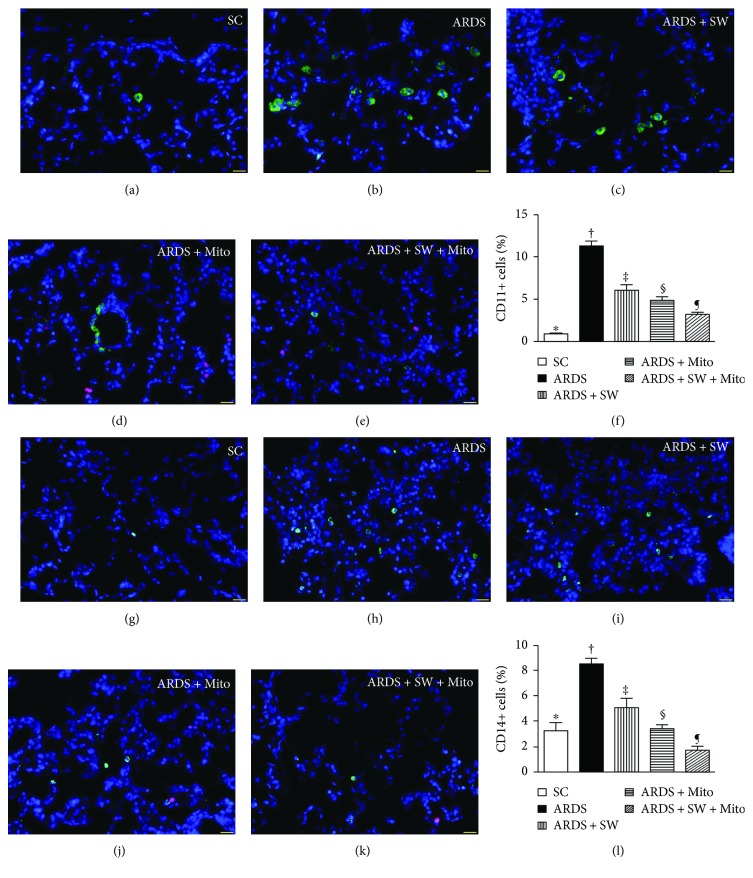
CD11+ and CD14+ cell infiltration in lung parenchyma by day 5 after ARDS induction. (a to e) Immunofluorescent (IF) microscopic finding (400x) for identification of CD11+ cells (green color) in lung parenchyma. Red color indicated exogenous mitochondria. (f) Analytical result of number of CD11+ cells, ^∗^ versus other groups with different symbols (†, ‡, §, ¶), *p* < 0.0001. (g to k) IF microscopic finding (400x) for identification of CD14+ cells (green color) in lung parenchyma. Red color indicated exogenous mitochondria. Analytical result of number of CD14+ cells, ^∗^ versus other groups with different symbols (†, ‡, §, ¶), *p* < 0.0001. The scale bars in the right lower corner represent 20 *μ*m. All statistical analyses were performed by one-way ANOVA, followed by Bonferroni multiple comparison post hoc test. Symbols (^∗^, †, ‡, §, ¶) indicate significance (at 0.05 level). SC = sham control; ARDS = acute respiratory distress syndrome; Mito = mitochondria; SW = shock wave.

**Figure 6:**
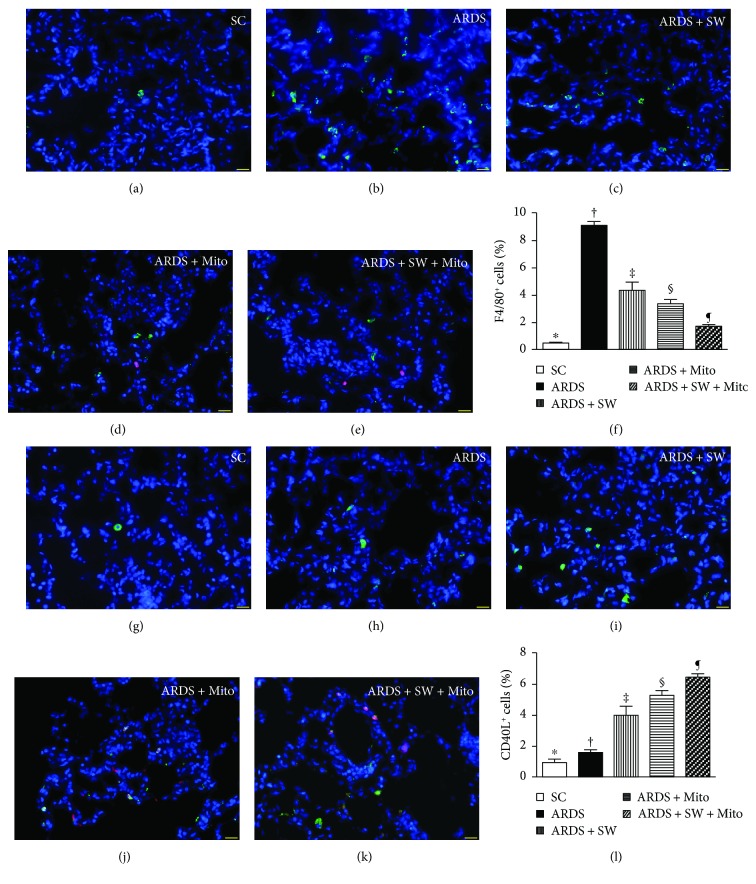
F4/80+ and CD40L+ cell infiltration in lung parenchyma by day 5 after ARDS induction. (a to e) Immunofluorescent (IF) microscopic finding (400x) for identification of F4/80+ cells (green color) in lung parenchyma. Red color indicated exogenous mitochondria. (f) Analytical result of number of F4/80+ cells, ^∗^ versus other groups with different symbols (†, ‡, §, ¶), *p* < 0.0001. (g to k) IF microscopic finding (400x) for identification of CD40L+ cells (green color) in lung parenchyma. Red color indicated exogenous mitochondria. (l) Analytical result of number of CD40L+ cells, ^∗^ versus other groups with different symbols (†, ‡, §, ¶), *p* < 0.0001. The scale bars in the right lower corner represent 20 *μ*m. All statistical analyses were performed by one-way ANOVA, followed by Bonferroni multiple comparison post hoc test. Symbols (^∗^, †, ‡, §, ¶) indicate significance (at 0.05 level). SC = sham control; ARDS = acute respiratory distress syndrome; Mito = mitochondria; SW = shock wave.

**Figure 7:**
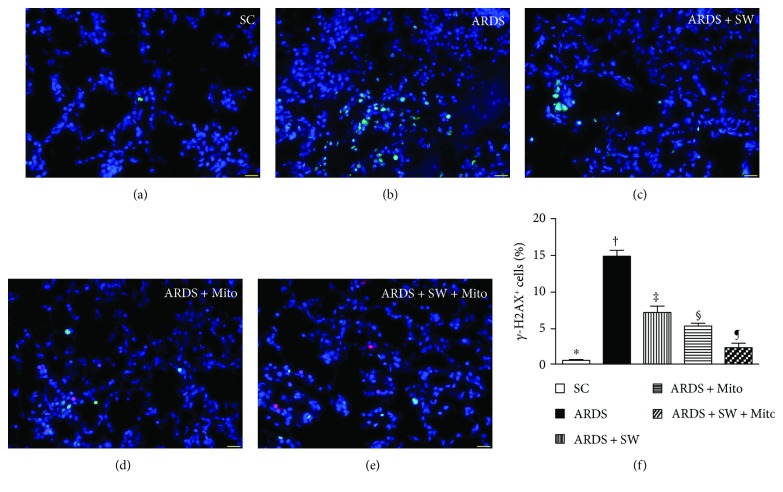
DNA damage marker in lung parenchyma by day 5 after ARDS induction. (a to e) Immunofluorescent (IF) microscopic finding (400x) for identification of *γ*-H2AX+ cells (green color) in lung parenchyma. Red color indicated exogenous mitochondria. (f) Analytical result of number of *γ*-H2AX+ cells, ^∗^ versus other groups with different symbols (†, ‡, §, ¶), *p* < 0.0001. The scale bars in the right lower corner represent 20 *μ*m. Symbols (^∗^, †, ‡, §, ¶) indicate significance (at 0.05 level). SC = sham control; ARDS = acute respiratory distress syndrome; Mito = mitochondria; SW = shock wave.

Immunofluorescence microscopy showed that 5 days after ARDS induction, infiltration of the lung parenchyma by CD11+, CD14+, F4/80+, and CD40L+ inflammatory cells was the highest in untreated ARDS rats, was significantly and progressively lower across the ARDS + SW, ARDS + Mito, and ARDS + SW + Mito groups, and was the lowest in SC rats (Figures 5 and 6). Immunofluorescence microscopy also revealed that the number of positively stained *γ*-H2AX cells, an indicator of DNA damage, exhibited a pattern identical to that of inflammatory cells among the five groups (Figure 7).

### 3.5. Histopathological Findings after ARDS Induction

**Figure 8:**
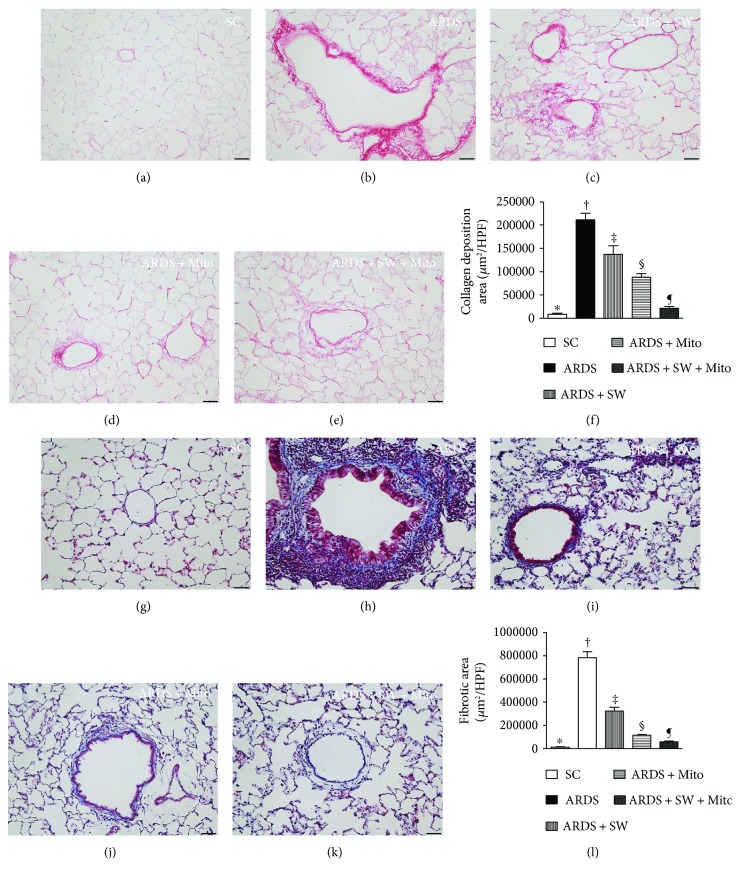
Histopathological findings of lung parenchyma by day 5 after ARDS induction. (a to e) Illustrating the microscopic finding (200x) of Sirius red stain for identification of condensed collagen deposition in lung parenchyma. (f) Analytical result of condensed collagen deposition area, ^∗^ versus other groups with different symbols (†, ‡, §, ¶), *p* < 0.0001. (g to k) Illustrating the microscopic finding (200x) of Masson's trichrome stain for identification of fibrosis in lung parenchyma. (l) Analytical result of fibrotic area, ^∗^ versus other groups with different symbols (†, ‡, §, ¶), *p* < 0.0001. The scale bars in the right lower corner represent 50 *μ*m. All statistical analyses were performed by one-way ANOVA, followed by Bonferroni multiple comparison post hoc test. Symbols (^∗^, †, ‡, §, ¶) indicate significance (at 0.05 level). SC = sham control; ARDS = acute respiratory distress syndrome; Mito = mitochondria; SW = shock wave.

Five days after ARDS induction, sections stained with Sirius oil red exhibited areas of condensed collagen deposition indicative of lung parenchymal damage with activation of fibroblasts and deposition of collagen fibers (Figure 8). Staining was the highest in specimens from untreated ARDS rats, the lowest in SC, significantly higher in the ARDS + SW and ARDS + Mito groups than in the ARDS + SW + Mito group, and significantly higher in the ARDS + SW than in the ARDS + Mito group. Additionally, Masson's trichrome staining revealed that in all five groups, the fibrotic areas displayed identical patterns of collagen deposition.

### 3.6. Oxidative Stress in Lung Parenchyma after ARDS Induction

**Figure 9:**
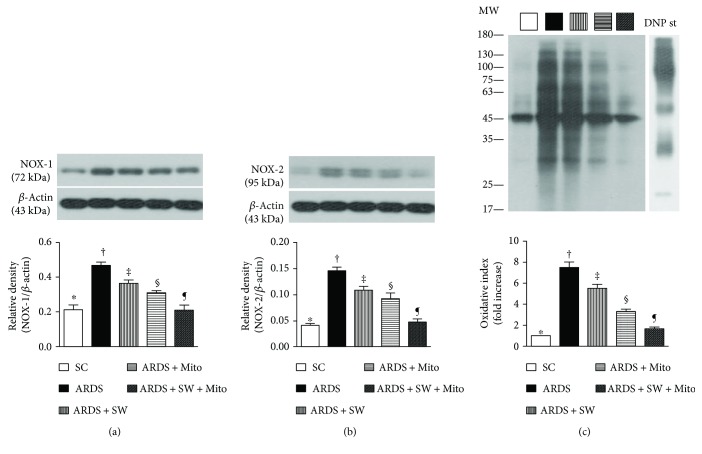
Protein expressions of oxidative stress in lung parenchyma by day 5 after ARDS induction. (a) Protein expressions of NOX-1, ^∗^ versus other groups with different symbols (†, ‡, §, ¶), *p* < 0.0001. (b) Protein expression of NOX-2, ^∗^ versus other groups with different symbols (†, ‡, §, ¶), *p* < 0.0001. (c) Expressions of oxidized protein, ^∗^ versus other groups with different symbols (†, ‡, §, ¶), *p* < 0.001. MW = molecular weight; DNP = 1,3-dinitrophenylhydrazone. All statistical analyses were performed by one-way ANOVA, followed by Bonferroni multiple comparison post hoc test. Symbols (^∗^, †, ‡, §, ¶) indicate significance (at 0.05 level). SC = sham control; ARDS = acute respiratory distress syndrome; Mito = mitochondria; SW = shock wave.

Five days after ARDS induction, levels of NADPH oxidase- (NOX-) 1, NOX-2, and oxidized protein, three indicators of oxidative stress, were the highest in untreated ARDS rats, were significantly and progressively reduced across the ARDS + SW, ARDS + Mito, and ARDS + SW + Mito groups, and the lowest in the SC group (Figure 9).

### 3.7. Biomarkers of Inflammation in Lung Parenchyma after ARDS Induction

**Figure 10:**
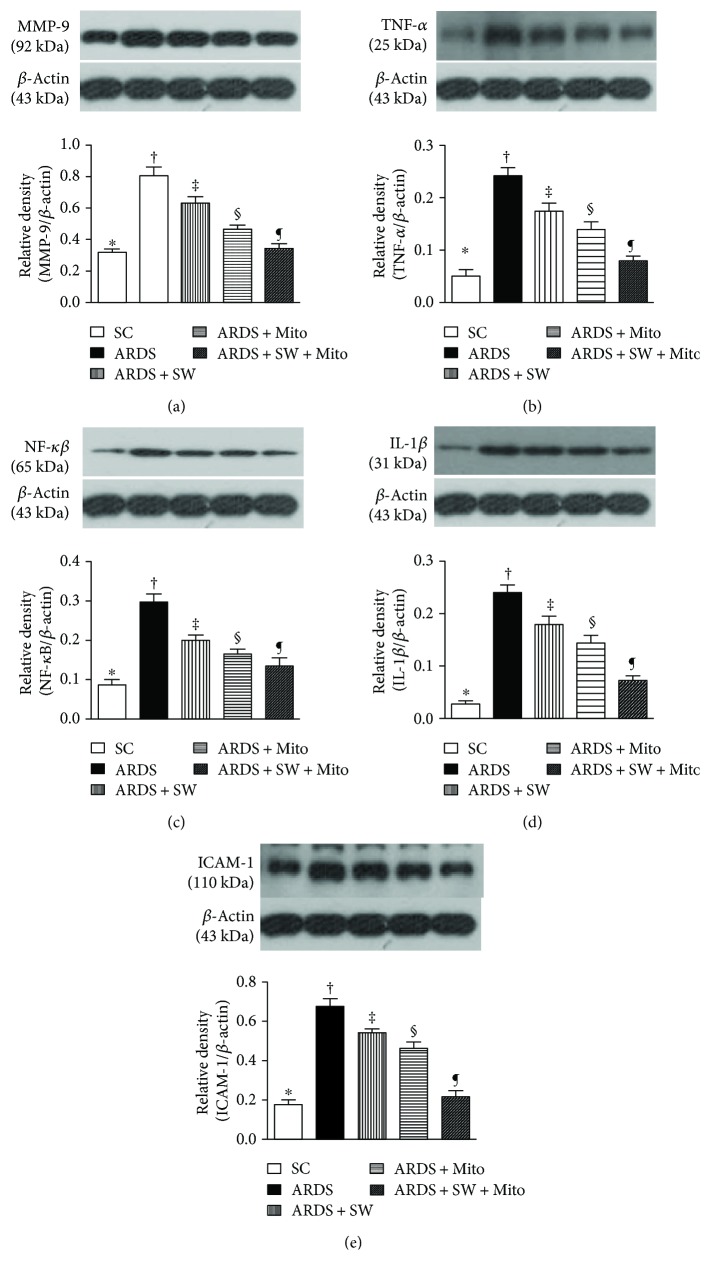
Protein expressions of inflammation in lung parenchyma by day 5 after ARDS induction. (a) Protein expression of matrix metalloproteinase- (MMP-) 9, ^∗^ versus other groups with different symbols (†, ‡, §, ¶), *p* < 0.0001. (b) Protein expression of tumor necrosis factor- (TNF-) *α*, ^∗^ versus other groups with different symbols (†, ‡, §, ¶), *p* < 0.0001. (c) Protein expression of nuclear factor- (NF-) *κ*B, ^∗^ versus other groups with different symbols (†, ‡, §, ¶), *p* < 0.0001. (d) Protein expression of interleukin- (IL-) 1*β*, ^∗^ versus other groups with different symbols (†, ‡, §, ¶), *p* < 0.0001. (e) Protein expression of intercellular adhesion molecule- (ICAM-) 1, ^∗^ versus other groups with different symbols (†, ‡, §, ¶), *p* < 0.0001. All statistical analyses were performed by one-way ANOVA, followed by Bonferroni multiple comparison post hoc test. Symbols (^∗^, †, ‡, §, ¶) indicate significance (at 0.05 level). SC = sham control; ARDS = acute respiratory distress syndrome; Mito = mitochondria; SW = shock wave.

Five days after ARDS induction, levels of matrix metalloproteinase- (MMP-) 9, tumor necrosis factor- (TNF-) *α*, nuclear factor- (NF-) *κ*B, interleukin- (IL-) 1*β*, and intercellular adhesion molecule- (ICAM-) 1, five indices of inflammation, were the highest in untreated ARDS rats, were significantly and progressively reduced across the ARDS + SW, ARDS + Mito, and ARDS + SW + Mito groups, and the lowest in the SC group (Figure 10).

### 3.8. Biomarkers of Apoptosis, Fibrosis, and Mitochondrial Damage in Lung Parenchyma after ARDS Induction

**Figure 11:**
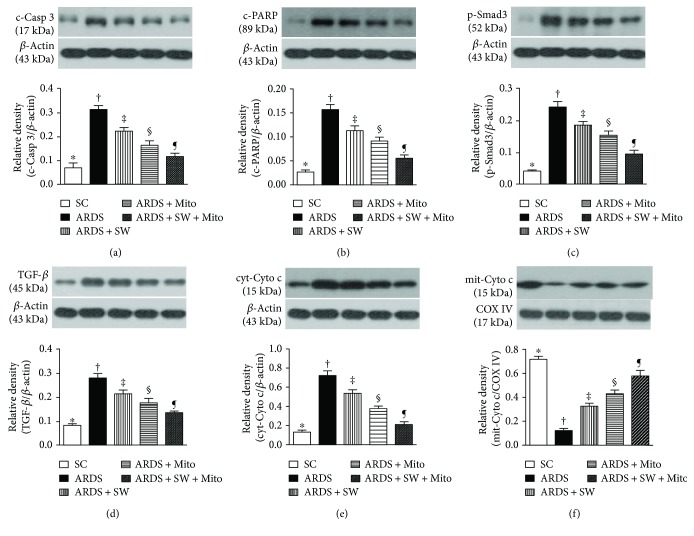
Protein expressions of apoptotic, fibrotic, and mitochondrial damage biomarkers in lung parenchyma by day 5 after ARDS induction. (a) Protein expression of cleaved caspase 3 (c-Casp 3), ^∗^ versus other groups with different symbols (†, ‡, §, ¶), *p* < 0.0001. (b) Protein expression of cleaved poly(ADP-ribose) polymerase (c-PARP), ^∗^ versus other groups with different symbols (†, ‡, §, ¶), *p* < 0.0001. (c) Protein expression of phosphorylated- (p-) Smad3, ^∗^ versus other groups with different symbols (†, ‡, §, ¶), *p* < 0.0001. (d) Protein expression of transforming growth factor- (TGF-) *β*, ^∗^ versus other groups with different symbols (†, ‡, §, ¶), *p* < 0.0001. (e) Protein expression of cytosolic cytochrome c (cyt-Cyto c), ^∗^ versus other groups with different symbols (†, ‡, §, ¶), *p* < 0.0001. (f) Protein expression of mitochondrial cytochrome c (mit-Cyto c), ^∗^ versus other groups with different symbols (†, ‡, §, ¶), *p* < 0.0001. All statistical analyses were performed by one-way ANOVA, followed by Bonferroni multiple comparison post hoc test. Symbols (^∗^, †, ‡, §, ¶) indicate significance (at 0.05 level). SC = sham control; ARDS = acute respiratory distress syndrome; Mito = mitochondria; SW = shock wave.

Levels of cleaved caspase 3 and cleaved poly (ADP-ribose) polymerase (PARP), two indicators of apoptosis, were the highest in untreated ARDS, significantly and progressively reduced across the ARDS + SW, ARDS + Mito, and ARDS + SW + Mito groups, and the lowest in the SC groups. The same pattern was exhibited by Smad3 and transforming growth factor- (TGF-) *β*, two mediators of fibrosis, as well as cytosolic cytochrome c, an indicator of mitochondrial damage. Conversely, the opposite pattern was exhibited by mitochondrial cytochrome c, an indicator of mitochondrial integrity (Figure 11).

## 4. Discussion

In this study, we investigated the ability of SW plus mitochondrial therapy to protect the lung parenchyma from ARDS injury. Our main findings are that (1) transfused mitochondria are taken up into rat lung epithelial and parenchymal cells, (2) this effect is enhanced by SW therapy, and (3) SW plus mitochondrial therapy alleviates inflammation and ARDS damage within the lung parenchyma. Our finding that transfused, liver-derived mitochondria are taken up into lung parenchymal cells in ARDS rats is consistent our previous study [30]. Moreover, to our knowledge, this is the first report that SW therapy enhances that effect.

Mitochondrial therapy also appears to mitigate ischemia-reperfusion injury in rat liver [26, 28]. Furthermore, we previously showed that SW therapy ameliorates ischemia-related tissue fibrosis/ischemia-related organ damage and reduces ischemia-related organ dysfunction [34, 36, 37]. Interestingly, our histopathological findings show that ARDS-related lung fibrosis and condensed collagen deposition are greatly attenuated by SW therapy and that those beneficial effects are enhanced by mitochondrial therapy. We also observed that biomarkers of inflammation, DNA/mitochondrial damage, apoptosis, and fibrosis are all markedly elevated in ARDS animals, which is consistent with our earlier studies [30, 35]. Importantly, these parameters are all reduced by SW or mitochondrial therapy and further ameliorated by the combination of the two therapies. This extends our earlier findings [26, 28–30, 34, 36, 37] and may explain why lung injury scores (i.e., increased septal thickness and decreased number of alveolar sacs) and exudate leakage from lung parenchyma are reduced in ARDS animals treated with SW plus mitochondrial therapy.

SW therapy is widely accepted for clinical application for treatment of lithotripsy [38] as well as skeletal diseases [39, 40], ischemic heart disease [41, 42], and peripheral artery disease [43, 44]. The mechanisms underlying the beneficial effects of SW therapy are thought to be related to its anti-inflammatory [32–34, 45] and angiogenic effects [32–34, 46], which enhance the healing process [47]. On the other hand, the mechanism by which SW therapy enhances transport of mitochondria into cells remains unclear. Perhaps, it induces a transient increase in cell membrane permeability, leading to increased mitochondrial uptake into the cells. Consistent with that idea, previous studies showed that SW therapy increased cellular permeability, thereby facilitating delivery of genes and other molecules into cells [48, 49]. In addition, when exogenous mitochondria are taken up into cells, they often fuse and function with endogenous mitochondria (refer Figure 1). This could, at least in part, explain why SW plus mitochondrial therapy is superior to SW or mitochondrial therapy alone for protecting the lung against ARDS-induced damage.

This study has limitations. First, although the short-term outcome was promising, the long-term outcome of SW plus mitochondrial therapy for ARDS is unclear. Second, without assessment of the optimal doses of SW and mitochondrial therapy for ARDS, the relative efficacies of SW and mitochondrial therapy are uncertain.

## 5. Conclusions

In summary, the present study shows that SW therapy enhances uptake of mitochondria into lung epithelial and parenchymal cells and that SW-assisted mitochondrial therapy offers additional protection to the lung architecture against ARDS-induced injury.

## Data Availability

The data used to support the findings of this study are available from the corresponding author upon request.

## Abbreviations

ARDS: Acute respiratory distress syndrome
BAL: Bronchoalveolar lavage
c-Casp 3: Cleaved caspase 3
c-PARP: Cleaved poly(ADP-ribose) polymerase
cyt-Cyto c: Cytosolic cytochrome c
ETC: Electron transport chain
IF: Immunofluorescent
ICAM: Intercellular adhesion molecule
IL: Interleukin
LEC: Lung epithelial cells
MMP: Matrix metalloproteinase
Mito: Mitochondria
mit-Cyto c: Mitochondrial cytochrome c
NOX: NADPH oxidase; mitochondria
NF-*κ*B: Nuclear factor-*κ*B
OXPHOS: Oxidative phosphorylation machinery
OCR: Oxygen consumption rate
ROS: Reactive oxygen species
SC: Sham control
SW: Shock wave therapy
TGF: Transforming growth factor.
